# Supplementary material for: Are carbon emissions trading and green financial instruments synergistic? -Comprehensive quantitative research based on content analysis
Source: PLoS One. 2024 Mar 7;19(3):e0298601. doi: 10.1371/journal.pone.0298601 (PMC10919601; doi:10.1371/journal.pone.0298601)
Supplement: S1 Data — (ZIP) [file pone.0298601.s001.zip › Data/地级市Z_F工作报告.zip!/║■─╧/╓Ω╓▐╩╨/└┤╫╘╣1⁄2╓┌║┼í╛┬φ┐╦╩2╛▌═°í┐_2014.docx]

各位代表：

　　现在，我代表市人民政府向大会报告工作，请予审查，并请各位政协委员和其他列席人员提出意见。

　　一、2013年工作回顾

　　2013年是不平凡的一年，面对复杂多变的国内外经济形势、面对经济下行的巨大压力、面对严重的旱涝灾害，在省委、省政府和市委的坚强领导下，我们团结和依靠全市人民，紧扣“奋力打造株洲发展升级版”工作主题，顺势而谋、聚势而为、真抓实干、攻坚克难，全市经济实现了平稳健康发展，社会大局和谐稳定。预计全年实现GDP1960亿元，增长10.5%；完成公共财政预算总收入235.6亿元，增长10.2%；完成固定资产投资1500亿元，增长30%；完成社会消费品零售总额659亿元，增长13.6%；城镇居民人均可支配收入达到28663元，农民人均纯收入达到12398元，分别增长10.6%和13%。

　　一年来，我们突出抓了以下五个方面的工作：

　　（一）突出产业转型，发展质量稳步提升

　　新型工业化强力推进。不断强化工业主导地位，加速推进新型工业化工作继续保持全省先进行列。优势产业支撑作用进一步凸显，轨道交通产业产值增长13.5%，全市规模工业增加值增长12.5%，规模以上工业企业实现净利润增长17.5%。企业自主创新能力进一步增强，承担国家重大科技专项、国家863等重大科技计划项目15项，轨道交通装备制造业列入国家首批创新型产业集群试点。成功获批国家知识产权示范城市，第九次获评国家科技进步先进城市，高新技术产业增加值增长16%。园区实力进一步提升，建成标准厂房100万平方米，园区技工贸总收入增长18.5%。非公经济发展进一步加快，非公经济增加值增长11.5%，占GDP比重达到57%，连续三年获评全省非公经济和中小企业发展考核一等奖。建筑业实现总产值410亿元，增长23.5%。

　　现代服务业加速提升。出台了加快第三产业发展的若干意见，明确了发展重点，强化了政策措施，产业发展水平得到进一步提升。大力发展金融服务业，民间金融中心挂牌设立，农信社改革顺利推进，引进和新组建各类金融机构9家。全面提升文化旅游业，华强方特二期建设加速推进，攸州森林公园升格为国家级森林公园，茶陵县城创建为省级历史文化名城，炎帝陵获批为全国海峡两岸交流基地。旅游总人数达到2735.7万人次，实现旅游总收入171.6亿元，分别增长22.1%、22.4%。培育壮大现代物流业，A级物流企业达到9家，芦淞服饰物流配送中心等重点项目建设扎实推进。房地产业平稳健康发展。成功获批国家首批智慧城市试点、国家下一代互联网示范城市和信息消费示范城市。福布斯最佳商业城市排名由第72位前移到第68位。

　　农业现代化加快发展。坚持农业机械化、规模化、产业化、品牌化发展方向，全面提升农业现代化水平。着力发展规模农业，农业增加值增长3%，农民专业合作社增加到1023家，集体土地流转率提高到36%，生猪规模化养殖比重达到68%，城镇蔬菜新扩基地1万余亩。着力发展加工农业，省级以上农产品加工龙头企业达到21家，农产品加工总产值增长16%。竹木加工、花卉、油茶产业化步伐加快。着力发展科技农业，水稻机械化水平达到66.4%。着力发展品牌农业，新增省著名商标、市级以上品牌53个，麻鸭、白鹅、黄牛等特色农产品的品牌效应不断扩大。

　　县域经济持续壮大。坚持分类指导，培育特色产业，提升县域综合实力。经济总量稳步增长，县域经济总量达到989亿元，增长10.6%，占全市经济总量的比重提高到50.5%。综合实力继续增强，醴陵市在全国百强县排名上升到第86位，并入选全省首批特色县域经济重点县，攸县连续8年获评全省十强县。产业特色日益突出，株洲县的新能源、新材料、电子信息技术产业初具雏形，茶陵县建材产业园和建筑陶瓷产业园初具规模，并被确定为“全国陶瓷产业转移承接示范基地”，炎陵县形成了以神农谷、炎帝陵、红色革命旧址为重点的旅游产业格局。

　　（二）突出项目建设，发展后劲明显增强

　　项目推进力度加大。全力组织“项目大会战”，全年新开工项目总投资953亿元，增长39.1%。旗滨玻璃搬迁进展顺利，新芦淞服饰产业园、高精传动、山河智能等项目加快推进，IGBT产业化、中航通发航空发动机维修中心、海纳川株洲零部件基地、达沃斯行政总部园等项目相继竣工投产。

　　项目引进成果丰硕。加强招商引资，积极参加国、省系列活动，成功举办深圳招商引资推介会，成功承办第六届湘商大会，成功签约了一大批重大项目。全年引进资金442亿元，增长26%。加强项目对接，微软创新中心及产业园、北汽二工厂30万辆汽车等项目相继落户株洲，进驻株洲的世界500强企业新增2家，央企新增8家。加强争资立项，向国家和省里成功争取了一批重大项目，清水塘列入全国首批老工业区搬迁改造试点，G320国道莲易段改建高速正式获批。

　　要素保障坚强有力。全力破解融资和用地难题，“百日攻坚”和“再攻坚”行动完成征地拆迁交地2.2万亩、国有土地上房屋征收26.6万平方米，征拆征收量为2012年的2倍，有力地解决了一批重大项目的落地问题。金融支撑作用明显，保险、证券、期货市场稳步规范发展，组织了三次银企洽谈，对接项目201个，争取银行信贷授信支持324亿元，城发、天易、国投集团获批企业债券40亿元。煤电油气等其他生产要素支撑全面加强。

　　（三）突出统筹发展，城乡面貌持续改观

　　规划引领作用加强。强化规划引领发展，进一步完善了各类规划。在宏观上注重顶层设计，第六次城市总体规划（2006-2020年）修编已通过省部专家评审并呈报国务院，制定了到2030城市总规展望等战略规划，“一区两片、两轴四带”城镇结构体系初见雏形，编制了南部新城和东部新城概念性规划。在中观上注重提升品质，完成了城市发展空间结构、城市景观风貌、城市公共交通体系等规划编制。在微观上注重规划落实，形成了一批较高质量的控制性详规和城市设计，全面启动了乡镇村庄规划编制。同时，高质量完成了“十二五”规划中期评估。

　　交通条件明显改善。坚持交通优先发展，交通基础建设取得新突破，市域交通发生格局性变化，方便快捷的市域交通圈初步形成。全年完成交通基础设施建设投资140亿元，醴茶高速、垄茶高速、炎汝高速株洲段、衡茶吉铁路（货运）全线通车，新增高速公路通车里程207公里，G106茶炎段改造、铜塘湾港区一期、湘江2000吨级航道疏浚全面完工，长株潭城际铁路、沪昆高铁株洲段、湘江六桥建设加快推进，荷塘大道、神农大道核心段、中环东路北段基本建成。完成农村公路“通畅工程”、“连通工程”326公里，建成乡镇客运站、农村客运招呼站210个，建制村通畅率100%。成功获批创建国家“公交都市”示范城市。

　　城乡环境不断变美。坚持城乡一体化发展方向，深入推进城乡环境同治，城乡环境进一步改善。加大创建力度，顺利通过国家卫生城市复审、全国交通管理模范城市复评、全国文明城市“三项测评”，攸县、茶陵成功创建国家卫生县城，“仁达大厦”实现我市“鲁班奖”零的突破。推进国家森林城市创建，森林覆盖率达到61.9%。加强环境整治，启动或完成湘江流域重金属治理项目45个，关停重金属污染企业51家，拆除烟囱34根，节能减排完成年度目标任务。深入推进“一江四港”综合整治，白石港水质净化中心投入试运行，二水厂提质改造工程全面完成，龙泉污水处理厂三期基本建成，霞湾港重金属污染治理成效明显，湘江株洲段水质保持Ⅲ类以上，市区集中式饮用水源水质达标率100%。加速两型示范区建设，云龙示范区着力打造“森林田园城市”，获评国家绿色生态示范城区；两型项目建设凸显特色，连片20兆瓦光伏发电、华新水泥窑协同处理生活垃圾等项目成为全省两型典范。加快农田水利建设，除险加固病险水库89座，整治河道堤防51公里，建设各类水利工程3万余处，解决了16.3万农村人口饮水安全问题，“节水型”社会试点通过国家验收。

　　（四）突出改革开放，发展活力不断释放

　　重点改革实现新突破。出台了株洲市经济体制改革重点工作的意见，启动了21项专项经济体制改革，“个转企”1453户，“小升规”313家。加大简政放权力度，出台《关于促进产业园区发展升级的若干意见》，下放园区市级经济管理权限62项。创新土地管理制度，基本完成农村集体土地所有权确权登记工作，成为全省首个使用耕保“一张图”系统的地级市。深化财税体制改革，税收“营改增”改革试点正式启动，预决算公开试点工作有序推进。

　　两型改革取得新成效。探索创立了“一县（市）区一主题，一部门一专项”的改革组织模式，建立了改革策划、评审（风险控制）批复、组织实施的工作机制。全面推进生活用水等资源性产品阶梯式价格改革；率先实施建设项目环境、能耗、水土保持等评价的综合评审制度；建立了大气污染联防联控和应急处理机制，实行了重点企业环保信息公开；建立了集体土地使用权抵押融资机制，扩大了集体建设用地的权益和功能。

　　开放合作迈出新步伐。加快培育外向型企业，新增外贸实绩企业45家、国际电子商务平台企业95家。大力拓展新兴市场，产品出口到145个国家和地区，完成外贸进出口总额25.1亿美元，增长16.8%。积极实施“走出去”战略，南车株机在深圳获得20亿元地铁订单的同时，在南非设立公司，获得4亿美元机车订单。

　　（五）突出民生改善，发展成果更加惠民

　　社会保障继续加强。坚持社会基本保障托底，涉及民生的财政支出达169亿元，占公共财政预算支出的65.7%。为民办实事目标任务全面或超额完成。积极推进就业创业，湖南（株洲）就业创业指导中心建设加快推进，新增城镇就业5.96万人，新增农村劳动力转移就业5.3万人，失业人员再就业2.6万人，城镇零就业家庭动态援助达100%。继续扩大社会保险覆盖面，新增企业养老保险参保4.8万人，新型农村养老保险参保率达92%，城镇基本医疗保险实现全覆盖，新农合参合率达98.7%。全力保障城乡困难群众基本生活，城区居民最低生活保障标准提高到400元，城区最低工资标准提高到1265元，“失独”家庭特别扶助金每月提高到300元，农村五保老人集中供养率达到36%，均居全省领先水平；城镇低保保障7.7万人，农村低保保障11.6万人，城区一、二级残疾人享受重残补贴。切实加强保障性住房建设，开工建设廉租房1912套、公租房9431套，改造棚户区7506户、农村危房3570户；发放住房公积金贷款19.5亿元，支持居民购房、建房82万多平方米，城乡居民的居住条件进一步改善。

　　社会事业全面进步。坚持教育强市目标不动摇，69项测评指标已达标65项，达标率94.2%，建设合格学校117所，茶陵、炎陵等6县市区通过国家义务教育发展基本均衡县验收；新增普惠幼儿园343所，建设和管理经验获全国教育改革创新特别奖；职教科技园加快建设，湖南商业技术学院和湖南铁路科技职业技术学院建成开学。加强医疗卫生服务体系建设，公立医院绩效分配制度改革试点全面实施，取消“以药补医”综合配套机制改革全面启动，市中心医院整体搬迁投入使用，32个乡镇卫生院标准化建设全面完成。加大文化惠民力度，神农大剧院、神农艺术中心进展顺利，炎帝陵神农园项目启动建设，“乡村大舞台”成功申报为全国公共文化服务示范项目，国家重点文物保护单位增加7处。成功举办市十二届运动会，群众文体活动丰富多彩。人口和计生工作成功返省模。与此同时，妇女儿童、老龄、残联、机构编制、外事侨务、广播电视、国家安全、工商、质监、海关、检验检疫、供销、贸促、人防、气象、水文、统计、市志、档案、民族宗教、红十字、对口支援、移民、志愿者服务等工作都有新进步。

　　社会大局和谐稳定。深化“平安株洲”建设，综治工作民调满意率名列全省前茅，荣获全省社会管理综合治理先进市，第三次捧得全国“长安杯”。全面开展安全生产大检查，认真落实各项安全生产责任，安全生产形势平稳可控。大力开展食品药品安全专项整治，确保了人民群众饮食用药安全。深入开展双拥共建活动，全面加强国防动员和民兵预备役工作，驻株解放军、武警部队和民兵预备役人员积极遂行抗旱救灾、抗洪抢险、维稳处突等急难险重任务，为株洲经济社会发展作出了重要贡献。

　　一年来，我们围绕建设“四型”政府，切实加强政府自身建设。认真贯彻落实中央、省委和市委关于作风建设的一系列规定，深入开展“优环境、提效能、促升级”活动，注重解决突出问题，机关作风进一步转变，行政效能和服务水平有新的提升。注重制度建设，修订出台《株洲市人民政府工作规则》，新出台《株洲市政府投资管理办法》，规范行政行为。认真执行人大及其常委会的决议决定，自觉接受人大、政协和社会各界的监督，进一步促进了政府决策科学化、民主化、法制化；认真办理人大代表建议、政协委员提案，见面率、办结率、满意率均为100%。认真处理市长热线和市长信箱中的群众咨询、建议和投诉，共处理5.9万件，办结率为99.3%。加大了审计监督和行政监察力度，严格落实廉政建设责任制，强化责任追究，推动了清廉行政。

　　各位代表！过去的一年，发展的成果令人鼓舞，成绩来之不易。这是省委、省政府和市委坚强领导的结果，是市人大、市政协监督支持的结果，是全市上下同舟共济、奋力拼搏的结果。在此，我代表市人民政府，向全市广大工人、农民、干部、知识分子和企业家，向各民主党派、工商联、无党派人士、各人民团体，向离退休老领导、老同志，向驻株部队、武警官兵、公安政法干警，向所有关心支持株洲发展的社会各界朋友，表示崇高的敬意和衷心的感谢！

　　在总结成绩的同时，我们也清醒地看到，我市经济社会发展还存在一些亟待解决的困难和问题：一是经济增长的动力还不足。重大项目储备不多，投资接续能力不强，消费增长乏力，出口贸易不畅，对经济增长的支撑作用不强。二是经济转型升级任务艰巨。部分传统产业的优势逐渐弱化，新的替代产业尚未壮大，规模工业在全省排位后移，第三产业仍然是短板。三是城乡统筹任务繁重。区域发展不均衡，由北向南城市辐射带动能力依次减弱。县域经济的总量偏小，均量偏低，低收入人群依然不少。四是生态环境建设仍需加强。部分地区的工矿污染、农业面源污染，以及水、大气、土壤等污染治理任务还较重。五是发展环境有待优化。政府与市场的关系还有待理顺。行政审批项目和环节过多，行政效能不高，“四风”问题和不作为、缓作为、乱作为的现象还不同程度地存在。服务环境、法制环境、市场环境均有待改善。六是公共服务的提供水平与人民群众的期望仍有差距。对这些困难和问题，我们将高度重视，采取有力措施，切实加以解决。

　　二、2014年的总体部署和主要任务

　　2014年，是全面贯彻落实党的十八届三中全会精神、全面深化改革的首战之年，也是完成“十二五”规划目标的攻坚之年。当前，世界经济仍将延续缓慢复苏态势，存在许多不稳定不确定因素，新的增长动力源尚不明朗。我国经济处于控产能、去杠杆的换挡调整期，经济运行下行压力仍然不小。与此同时，我国经济发展长期向好的基本面没有变，中高速增长的态势没有变。我们也必须看到，推动株洲发展升级，我们还拥有十分难得的机遇：一是全面深化改革的行动已经铺开，改革的红利将进一步释放；二是国家支持产业转型升级、推进新型城镇化、开展罗霄山片区扶贫攻坚等政策实施将对我市产生诸多利好；三是株洲处于习总书记为湖南定位的“一部一带”的核心区，区位优势进一步凸现；四是沿海发达地区产业仍在加快转移，大力承接产业转移加快发展正当其时。面对新的发展形势，我们必须增强机遇意识，大胆解放思想，锐意改革创新，加速转型升级，增创新的优势，实现新的跨越。

　　2014年政府工作的指导思想是：全面贯彻落实党的十八大、十八届三中全会和省委、省政府和市委的决策部署，坚持稳中求进、好中求快的工作总基调，深入实施转型升级总战略，紧紧围绕“三个率先”的总目标，集中力量打好“四大攻坚战”，扎实开展“改革创新年、项目建设年、环境优化年”活动，着力激发市场活力，着力加快转方式调结构，着力保障和改善民生，着力创新社会治理，切实提高经济发展质量和效益，促进经济持续健康较快发展、社会和谐稳定。

　　今年全市经济社会发展的主要预期目标是：GDP增长11%；公共财政预算收入增长12%；全社会固定资产投资增长28%；社会消费品零售总额增长13.5%；城镇居民人均可支配收入增长11%，农民人均纯收入增长12%；万元GDP能耗下降3%，完成省里下达的年度减排任务；新增城镇就业人数5.2万人；人口自然增长率控制在9‰以内；各项事业都有新的发展。

　　与此同时，我们将集中力量抓好十大工业项目、十大农业产业化项目、十大服务业项目、十大基础设施项目、十大重点改革和民生100工程（详见附件）。

　　做好2014年各项工作，我们将牢牢把握“五个第一”：一是把转型发展作为第一要务。在发展要求上，更加注重发展质量，追求没有水分的、实实在在的发展，追求没有后遗症的、可持续的发展；在发展空间上，更加注重东向、南向发展，推动城乡统筹，加快县域崛起；在发展重点上，更加注重产业发展，做强工业，做大服务业，做优农业，努力构建现代产业体系。二是把改革创新作为第一动力。以改革创新总揽全局，坚持问题导向，全面梳理阻碍发展的体制机制问题，通过创新体制机制加以解决，激发发展的活力。三是把项目建设作为第一抓手。无论是产业发展，还是城乡建设，无论是改革创新，还是民生改善，所有的工作都不能只停留在思路上，都必须具体化为一个个项目，把工作抓落实、抓到位。四是把优化环境作为第一责任。政府是环境的创造者。要按照“标本兼治、综合治理”的原则，努力实现由重点加强基础设施建设向全面优化人居环境转变，由注重出台优惠政策向全面优化市场环境转变，由侧重维护治安秩序向全面优化社会环境转变。五是把改善民生作为第一追求。切实解决民生领域里的突出问题，切实办好涉及民生的大事和实事，把改善民生作为全部工作的出发点和落脚点，让株洲人民不断增强归属感和幸福感。

　　围绕上述指导思想和目标要求，今年，市政府将重点抓好以下八个方面的工作：

　　（一）着力推进改革攻坚，催生发展新动力。改革是做好各项工作的开路先锋。要用改革的精神、思路、办法来加快发展步伐，全面落实深化改革“规定动作”，积极探索株洲改革“自选动作”，不断释放改革红利，催生发展新动力。

　　全面深化经济体制改革。加快投融资体制改革。加强政府性债务管理，严格举债程序，防控债务风险；建立现代企业制度，加大市场化运作力度；由监管企业转为监管资本，引进社会资金参与基础设施和公用事业项目建设。稳步推进公共资源交易管理体制改革。建立统一的公共资源交易平台，完善公共资源交易监管体系。加快土地管理制度改革。完善城乡统一的建设用地市场，规范推进农村土地流转；推动不动产统一登记，完善农村集体土地确权登记发证管理体系，健全基层国土资源管理共同责任机制；规范用地管理，建立土地集约节约制度，加强土地储备管理，建立征地拆迁长效机制，继续开展征地拆迁攻坚战。加快财税体制改革。扩大预算公开试点范围，整合规范各类专项资金，加大预算绩效管理力度；全面推进 “营改增”试点工作。加快金融体制改革。认真落实金融“国十条”政策，推进城区、茶陵县、攸县、醴陵市、株洲县农信社改革工作，组建地方农村商业银行；加快炎陵县、攸县村镇银行组建步伐，推进荷塘区农村金融改革试点工作；进一步完善金融服务组织体系。加快企业改革。支持非公有制企业参与国有工业和商贸企业改革，大力发展混合所有制经济；加大“个转企”、“小升规”工作力度。

　　积极推动行政管理体制改革。推进政府机构改革。按照“精简、效能、统一”的原则和“三超两乱”专项治理的要求，严控机构编制，确保财政供养人员只减不增；积极推进卫生与计生行政部门机构和职责整合，完成省以下工商、质监管理体制调整和食品药品安全监管体制改革，加快推进医药卫生、司法、水务等方面改革涉及的体制调整工作。深化行政审批制度改革。创新联审联办审批模式，推进限时办结制度，强力推进“两集中，两到位”改革，确保网上审批和网上办结率均达到100%。最大限度取消和下放一批投资类、生产经营类、资质资格类等审批事项。统筹推进市政建设、维护等领域市场化改革，建立和完善政府向社会购买服务制度。加强对社会中介机构的清理整顿，建立健全监管制度，支持社会中介机构规范发展。顺势推进行政区划调整，更好地促进株洲发展。改革绩效考核办法，探索推行绿色GDP评价考核。

　　大力促进社会事业体制改革。稳妥推进事业单位分类改革，促进政事分开、事企分开、管办分离。深化教育领域综合改革。启动中小学教育质量综合评价改革试点工作，全面推进中小学教师职称制度改革，探索职业教育理事会管理模式，推动“地方政府促进高等职业教育发展改革试点”工作。深化医药卫生体制改革。深入推进公立医院改革，全面实施取消“以药补医”机制的综合配套改革，探索市区医疗资源整合新路径。深化文化体制改革。完善文艺院团法人治理结构，推进公益性文化事业单位劳动人事、收入分配和社会保障制度改革。

　　（二）着力推进工业振兴，增创工业新优势。放大株洲国有大中型企业的优势，放大现有传统产业的优势，充分利用国家促进产业转型升级的政策机遇，着力打造“五城四基地”，着力培育战略性新兴产业，着力提升全市工业发展水平。

　　加快工业转型升级。综合利用我市轨道交通、通用航空、汽车及零部件、风电等动力产业集聚优势，全力打造“中国动力谷”。加快建设轨道科技城、株洲通用机场、通用航空产业示范园、北汽二工厂、中国汽车零部件产业园、电动汽车、汽车主题公园等项目。加快建设醴陵陶瓷商贸城，发展壮大茶陵、攸县建筑陶瓷产业，推动陶瓷产业集群化发展。推动芦淞服饰市场群提档升级，抓好服饰电商城等项目建设，着力打造“中国·芦淞时尚服饰城”。加快有色冶金、化工等传统产业的改造升级，努力争取五矿集团相关事业部落户株洲。推进清水塘地区企业搬迁改造提升，以旗滨玻璃整体搬迁为契机，启动实施新的转型升级项目。建成攸县煤电一体化项目。大力发展电子信息、新能源、新材料、食品医药等战略性新兴产业，全力推进微软创新中心及电子信息产业园、阿里巴巴产业带、医学健康产业园等新兴产业园区项目建设，做大以千金药业为龙头的医药产业，壮大时代橡塑等非金属类新材料产业，推动具有比较优势的包装印刷产业发展。

　　引导企业集聚发展。全面落实《关于促进产业园区发展升级的若干意见》，深化园区“一权两制一司”改革，建立以项目落地为主的园区考评体系，激发园区发展活力。继续实施园区“五个一批”工程，加大力度储备一批配套到位的工业土地、引进一批企业、开工一批项目、建设一批标准厂房、做大一批投融资公司，新建标准厂房150万平方米，力争园区规模工业增加值占全市规模工业增加值的比重超过58%。加快优势产业集聚，延伸优势产业链条，强化国有大型企业对县域经济发展的辐射带动作用。

　　推动企业技术创新。以“创建国家创新型试点城市”为契机，重点实施“五个一” 工程，创建一批创新团队、实施一批重大专项、建设一批企业研发中心、打造一批协同创新平台、实施一批科技成果转化项目，推动企业加强技术改造和技术创新，确保新增高新技术企业20家以上，实现高新技术产业增加值450亿元以上。高标准建设国家知识产权示范城市，力争年专利申请量突破4000件。完善人才政策体系，用好本地人才，引进和培育高层次创新创业人才，打造人才高地。

　　加大企业服务力度。大力实施工业振兴“十百千”工程，加大对企业引导扶持力度，营造良好环境，着力培育一批过1亿元、10亿元、20亿元、50亿元、100亿元的优质企业。围绕轨道交通、汽车、航空等特色优势产业，引导企业加强产业协作配套，增强产业整体竞争能力。打破“玻璃门”、“弹簧门”，让非公经济与公有经济依法平等进入市场，鼓励非公经济快速发展。

　　（三）着力推进服务业升级，强化转型新支撑。加强引导扶持，全力推进服务业全面升级。

　　重点发展生产性服务业。围绕先进制造业，大力培育与之相配套的工业设计、现代物流、电子商务、服务外包等产业，拉长产业链，形成聚集效应。完善现代物流基础设施，积极引进大型网络营运商，促进现代交通、物流配送、电子商务体系和现代产业体系的对接、配套、融合。加快新芦淞商贸物流、普洛斯物流等重点项目建设。

　　大力发展生活性服务业。按照“满足生活需要、提升发展水平”的要求，大力发展住宿、餐饮、家政、养老等生活性服务业。突出抓好神农太阳城、神农文化休闲街、喜盈门家居广场等一批商业综合体项目。继续推进“万村千乡市场”工程，重点实施农贸市场改造，建设一批标准化农贸市场。

　　加快发展文化旅游服务业。重点围绕炎帝陵创国家5A级旅游景区，神农谷、云峰湖旅游度假区建设及云阳山、东阳湖、酒仙湖旅游综合开发，进一步完善旅游基础设施和配套设施，推进交通设施向旅游景区延伸，提升旅游服务质量。加快文化服务业发展，建成神农大剧院、神农艺术中心，推进醴陵世界陶瓷艺术城、中华茶祖文化产业园等重大项目建设。

　　（四）着力推进城镇扩容提质，构建全域新格局。坚持扩容与提质并重，高起点做好顶层设计，积极稳妥推进以人为核心的新型城镇化，加快形成城乡一体化发展格局，打造好湖南“东大门”。

　　优化城镇规划布局。规划布局“一区两片、两轴四带”开放型市域城镇空间架构，统筹推进株醴都市区、攸茶片区及炎陵片区的规划实施工作，形成中心城区、县城、中心镇功能互补、协调发展格局。加速城区“北联、南进、东拓”步伐，启动株洲东部新城和南部新城规划和建设，构建株醴发展轴和沿湘江城镇发展带，加快形成“一轴一带”的市区城镇发展格局。按照生产空间集约高效、生活空间宜居适度、生态空间山清水秀的总体要求，推进主体功能区建设，加快县城总体规划修订和村镇规划编制，少砍树、不填湖、少拆房，保留村庄原始风貌，真正把城镇放在大自然中，让居民望得见青山、看得见绿水、记得住乡情。

　　加强城镇基础设施建设。坚持交通引领城市发展理念，加快构建市域通畅的路网体系。建成沪昆高铁株洲段和醴陵东站，加快长株潭城际铁路、湘江六桥建设步伐，开工建设中环北路，湘江大道、渌枫大道、湘江七桥、株洲航电枢纽二线船闸、机场大道、武广客运枢纽站，规划建设东城大道，完善水陆综合交通体系。全面提升老旧社区、老旧厂区环境。推进城区自来水提质改造，加快研究城市第二水源建设问题。推行建筑垃圾综合利用、餐厨垃圾无害化处理，建成生活垃圾焚烧发电厂。加大城市管网建设和改造力度，实现城区雨污分流。

　　提升城镇管理水平。加强城市管理综合执法，强化市容市貌和绿化管理，推进垃圾直运管理。完善考核办法，加强各类创建活动，力争成功创建全国文明城市、国家环保模范城市；加快全国公交都市、国家森林城市创建步伐，推进健康城市建设。加大信息化建设整合力度，推进智慧株洲建设。深化交通秩序整治，重点治理红港路、建设南路、芦淞铁路涵洞等交通堵点，推动解决交通拥堵问题。加大卫生县城创建力度，尽快实现全域国家卫生城市目标。

　　（五）着力推进县域崛起，迈出小康新步伐。以率先全面建成小康社会为目标，按照“四化同步”、“五位一体”的总体部署，立足实际，突出特色，走出新路，让产业更强、居民更富、城乡更美。

　　提升农业现代化水平。加快土地流转和合作化经营，发展集约种植和规模养殖，切实加强主要农产品生产供给，确保粮食安全。紧盯“5年建成5个百亿产业”目标，切实建设粮油、养殖、竹木、禽畜水产品加工、饲料五大产业。实施品牌提升工程，切实推进农产品加工，壮大唐人神、好棒美、龙华农牧等龙头企业，年产值增长15%以上。推进中低产田改造、土地整理、农业面源污染防控、重金属污染治理和生态修复，完成30座小二型水库除险加固，切实提高农业综合生产能力。

　　加快工业化和城镇化进程。坚持项目支撑、园区突破，发展特色优势产业，积极承接产业转移，做大做强县域工业。以商贸流通、文化旅游、信息消费、住宿餐饮等为重点，放宽民营工商资本进入，加快发展县域服务业。支持县域扩容提质，建设一批特色明显、实力较强的工业强镇、商贸重镇、文化古镇、旅游名镇，构筑完善配套的城镇体系。加强路、电、水、通讯、能源建设，新（续）建、建成一批干线公路，改造一批危桥、渡改桥，实现公路、铁路、水路高效对接，构筑县域综合交通体系。加快农村客运站、招呼站和客运网线建设，改善出行条件。加快电网改造，完善通讯设施。强化农村环境综合整治，扎实推进美丽乡村建设，打造2个综合示范点，10个特色示范片，30个美丽乡村创建点。

　　推进基本公共服务均等化和扶贫开发攻坚。按照城乡一体化原则，着力提升县域居民文化、教育、医疗和社会保障等公共服务享有度。注重分类指导，推行差别化政策，实施精准扶贫。推进茶陵、炎陵两县规划实施，以项目建设促进产业发展、基础设施改善和教育进步。加强对口扶持，市里定向帮扶茶陵、炎陵的32个国家级贫困村，帮助增强“造血”功能，逐步缩小贫困地区发展差距，一定要让贫困地区居民同步提高生活水平、同步进入全面小康！

　　（六）着力推进两型建设，走出生态文明新路子。全力打好两型建设攻坚战，切实走出一条有别于传统发展方式的新路子来，在促进经济不断发展的同时，努力让株洲的水更绿，山更青，空气更清新。

　　切实促进资源节约利用。推广城镇土地立体开发，引导农村居民集中居住，盘活利用城乡存量建设土地，规范园区工业用地管理。加快重点行业节能改造，加强节能技术和产品的推广应用。实施居民用水阶梯价格制度，建设节水型城市。重视可再生能源开发和资源回收利用，重点开发利用太阳能、风能、地热能等清洁能源，推广建设工业园区厂房屋顶光伏电站。建立和完善废弃物回收利用系统；支持发展再制造业。

　　加强环境整治和生态保护。围绕省政府实施湘江流域保护与治理“一号工程”，启动湘江株洲城区河东段综合治理工程，整治好“一江四港”，完成“三个基本”行动年度目标任务，关停湘江城区段砂石厂，搬迁市区洗水企业，推进规模养殖退出城区。做好大气污染联防联控，推进低碳交通试点城市建设，强化机动车尾气排放控制，完成大唐华银脱硝改造等12个大气污染防治项目。保护好生态绿心。创建一批绿色示范集镇、示范村和社区。

　　突出示范片区引领作用。云龙示范区要走出一条既保护生态、又利用生态资本建设两型社会的路子，重点以创建国家智慧城区为契机，以引进微软创新中心及产业园为突破口，打造信息产业基地；统筹加快北部旅游休闲区、南部职教创新区等建设。天易片区要走出一条在新区建设两型社会的路子，重点加快两型产业集聚，大力推进栗雨中央商务区等项目建设。清水塘片区要走出一条在老工业区建设两型社会的路子，重点加大污染综合治理，加快重化工业绿色搬迁改造工作。全面开展两型集成与推广工作，把我市两型创建的成功经验进行系统集成，在面上推广。全面铺开12类集成创建活动，每个县市区形成一个以上两型综合示范片区，抓好“神农片区”省级两型综合示范片区创建。全面开展节能节水产品、清洁能源、环保建材、绿色出行等“十进片区”活动，大力开展两型特色县市区创建工作。

　　（七）着力推进对外开放，开辟发展新空间。发挥株洲处于“一部一带”核心区的区位优势，围绕打造好“湖南东大门”，在更大范围、更广领域、更高层次做好对外开放大文章，开辟发展新空间。

　　拓宽对外开放大通道。构建交通路网对外大通道，发展江海、铁海、路航等多式联运，形成对外经济走廊。全力申报株洲综合保税区、醴陵国家级经济技术开发区，加快铜塘湾港区建设，推动创建台资工业园，着力打造外向型经济发展大平台，使株洲真正成为人流、物流、资金流汇集和辐射的开放型区域中心。

　　推进对外开放大合作。加快引进来和走出去步伐，积极参加沪洽周等重要展会，主动承接长三角、珠三角、港澳台等经济发达地区产业转移，切实加强与世界500强、中国500强、国内行业龙头企业的项目对接，加快承接产业转移。不断拓展和深化友城合作，全力支持南车株机等企业拓展国际市场，加强合作交流。加强与周边城市、高铁沿线城市的交流合作，深入开展海峡两岸炎帝神农文化交流活动，加大经贸、文化、旅游等领域的对外合作力度。

　　优化对外开放大环境。制定促进对外开放的扶持政策，形成全方位开放的政策体系。建立健全政府、企业驻北京、上海、深圳等对外联络机构，完善产业、园区、平台的大招商推动机制。组建多部门联合的外经贸服务队，认真做好外向型企业“直通车”服务，提升服务开放发展的能力和水平。全面实行海关“属地申报、口岸验放” 和“无纸化通关”，全面推行检验检疫“绿色通道、直通放行”等便利措施，为承接产业转移、促进加工贸易创造良好环境。

　　（八）着力推进民生改善，开创和谐新局面。按照守住底线、突出重点、完善制度、引导舆论的思路，着力解决人民群众就业、就医、就学、住房等难题，努力增加居民收入，切实维护社会稳定，让人民群众生活更加幸福。

　　努力促进就业创业。把充分就业作为促进经济发展的优先目标，抓好高校毕业生、农村转移劳动力、城区困难人员和退役军人就业工作，实施离校未就业高校毕业生就业促进计划，做好企业下岗人员再就业工作。全年组织就业人才招聘会180场以上，新增城镇就业5.2万人，农村劳动力转移就业1.5万人。大力实施税费减免、创业补贴等扶持政策，加快工商登记制度改革，推进“百千万”工程和“返乡农民工创业”工程，发挥创业带动就业的倍增效应。全面启动就业信息系统建设，建成厅市共建湖南（株洲）就业创业指导中心。

　　不断强化社会保障。积极开展企业养老保险基金市级统收统支工作，实现城乡居民养老保险并轨。努力扩大参保缴费覆盖面，着力推进灵活就业人员、被征地农民、进城务工人员、重度残疾人等特殊群体的参保。全面实行城镇基本医疗保险和生育保险市级统筹，稳妥开展大病医疗互助和意外伤害引入商业保险工作，探索推进省内异地就医联网结算和跨统筹区保险关系转移接续工作。不断完善社会救助体系，重点保障好困难地区和困难群众。健全住房保障和供应体系，开工建设廉租房、公租房和改造各类棚户区1.9万套，建成1.6万套，发放廉租房租赁补贴1.5万户，进一步改善市民居住条件。

　　多途径增加居民收入。落实收入分配制度改革政策，着力扩大中等收入者比重，提高中低收入者收入水平，保持城乡居民收入合理增长，逐步缩小收入差距。落实惠民政策，多渠道增加居民财产性收入，逐步提高最低工资标准，完善工资支付保障制度，保护劳动所得，保护投资者合法权益。加强价格管理调控，保持物价基本稳定。

　　大力发展社会事业。坚持立德树人，加快建设教育强市步伐。统筹城乡义务教育资源均衡配置，实现义务教育向优质均衡迈进；促进学前教育向优质普惠升级，探索普通高中向特色化、多样化发展。推进合格学校建设，启动市特殊教育学校搬迁、市未成年人教育综合实践基地建设；加快职教科技园建设步伐，开工建设湖南铁道职业技术学院、湖南中医药高等专科学校，建成湖南化工职业学院。坚持以人为本，加快发展医药卫生事业。继续完善公共卫生服务体系，促进公共卫生服务均等化，切实防控重大传染病；优化医疗资源配置，加快推进市妇幼保健院搬迁及改扩建；启动实施临床医学重点专科建设三年行动计划，提升医疗服务水平。坚持文化惠民，推动文化事业全面繁荣。完成全市文化市场综合行政执法数字化平台建设，推进文化设施“四馆一宫一中心”建设，完成炎帝陵神农园主体工程。办好甲午炎帝陵祭祖大典。积极组团参赛省十二运会，大力推进全民健身运动。坚持计划生育基本国策，稳妥推进单独二孩政策落实，维护残疾人和妇女儿童权益，继续做好老龄、民族宗教、广播电视、对台、市志、档案、志愿者服务等工作。

　　维护社会和谐稳定。深入推进平安株洲、法治株洲建设，预防和严厉打击各种违法犯罪活动，维护社会公平正义；建立完善城市电子眼融合共享平台、公民信息平台和法治信访工作平台；健全完善社会稳定风险评估机制，及时解决群众合理诉求，加强“三调联动”，切实化解矛盾纠纷。完善突发事件应急管理机制，科学应对各种自然灾害和突发公共事件。加强隐患排查治理体系和安全预防控制体系建设，强力推进芦淞市场群消防隐患整改，坚决防止发生重特大安全事故。启动“智慧社区”建设。加强食品药品安全监管。健全人防防护体系，加强国防动员工作，推动军民共建深入开展。加强和创新宗教事务管理，争创民族团结进步模范集体六连冠。

　　三、切实加强政府自身建设

　　各位代表，做好2014年政府各项工作，责任重大，任务艰巨。我们将进一步加强政府治理体系和行政能力建设，全面提高政府公信力、执行力和创造力，努力打造人民满意政府。

　　（一）坚持转变职能，建设服务型政府。正确处理政府和市场的关系，充分发挥市场在资源配置中的决定性作用，切实把政府工作重点转到创造良好发展环境上来，转到提供优质公共服务上来，转到维护社会公平正义上来。要以开展好党的群众路线教育实践活动作为重要契机，以满腔的热情为广大企业排忧解难，以真挚的感情为人民群众多办实事，切实抓好民生100工程，坚决打击侵害企业和群众利益的行为，努力为企业营造一个良好的发展环境，为人民群众营造一个舒心的生活环境。

　　（二）坚持务实创新，建设效能型政府。深入开展“优环境、提效能、促升级”专项活动，注重务实创新，全面掀起“效能革命”，梳理并切实解决效能不高的突出问题。切实改进政府管理与服务方式，大力推进政务公开和电子政务，提升干部队伍素质，不断提高政府执行力。深化行政审批制度改革，没有法定依据的审批事项一律取消，重复审批的一律取消，暂时保留的一律规范提速，并更加注重事中事后监管。推行企业评议经济发展环境制度，积极探索建立政府绩效管理和考核模式，不断提高行政效能。

　　（三）坚持依法行政，建设法治型政府。加强法律学习和宣传，认真落实《株洲市法治政府建设规划（2013-2020年）》，深化行政执法体制改革，推行法律顾问制度。自觉执行人大及其常委会的决议、决定，接受人大及其常委会的法律监督、工作监督。支持和保障人民政协履行政治协商、民主监督和参政议政职能。政府出台重大决策前向本级人大报告，与政协协商。认真办理人大代表议案、建议和政协委员提案，广泛听取各民主党派、工商联、无党派人士和社会各界的意见。严格依照法定权限和程序行使权力，完善重大行政决策规则，把决策咨询、公众参与、合法性审查和集体讨论作为决策的必经程序，确保政府决策规范、科学、高效。

　　（四）坚持从严治政，建设廉洁型政府。严格执行党风廉政建设责任制和廉洁从政有关规定，认真执行党政机关厉行节约反对浪费条例，全面清理和规范公务用车、办公用房，全面规范公务接待，全面停止新建楼堂馆所。严格“三公”经费支出。自觉健全权力运行内部监督，强化决策程序管理，强化监察审计，着力从源头上预防和治理腐败。全面推进政务公开，确保权力在阳光下运行。

　　各位代表！我们正处在变革与奋进的伟大时代，梦想孕育奇迹，万事出自艰辛。空谈误国，实干兴邦。让我们在中共株洲市委的坚强领导下，解放思想、改革创新，万众一心、团结奋斗，在率先全面建成小康社会的新征程中，书写出无愧于伟大时代的壮丽诗篇！
